# A comprehensive survey of Rhinonyssid mites (Mesostigmata: Rhinonyssidae) in Northwest Russia: New mite-host associations and prevalence data

**DOI:** 10.3897/BDJ.8.e49535

**Published:** 2020-02-28

**Authors:** Manuel De Rojas, Jorge Doña, Ivan Dimov

**Affiliations:** 1 University of Seville, Seville, Spain University of Seville Seville Spain; 2 Illinois Natural History Survey, Champaign, United States of America Illinois Natural History Survey Champaign United States of America; 3 State Pediatric Medical University, St. Petersburg, Russia State Pediatric Medical University St. Petersburg Russia

**Keywords:** birds, checklist, ectoparasites, parasites, symbionts

## Abstract

**Background:**

Rhinonyssid mites are permanent parasites of birds that inhabit their respiratory tract. There are around 600 species described worldwide and almost all species of birds are found to have embedded rhinonyssid mites. Despite their presumed relevance, these mites are largely unstudied due to the difficulty in sampling them and, therefore, the majority of mite-host associations and species-prevalence data are unknown.

**New information:**

In this study, 179 mite specimens belonging to 27 species and eight genera were identified. Notably, 18 new mite-bird associations were documented for the first time, thus increasing the known host range for these mite species. In addition, mite-host associations found in this study were compared with known associations from these species of birds in the European part of Russia and in Europe. Overall, this study represents the largest survey to date carried out on rhinonyssid mites in Russia and one of the most comprehensive datasets on rhinonyssid host-range.

## Introduction

Nasal mites of the family Rhinonyssidae are permanent haematophagous endoparasites of birds that inhabit their respiratory tract (Vitzthum 1935; George 1961; Fain 1994; Dimov and de Rojas 2012). Most species live in the nasal cavity on the vascularised epithelial tissue; nevertheless, some species occupy the lungs, tracheal tissues and body cavity of their hosts (Lindquist et al. 2009; Krantz and Walter 2009). Rhinonyssids can not only cause damage to their hosts in a direct way (*Rhinonyssidosis avium disease*) (Dimov 2011), but could also be reservoirs or vectors of other infections like West Nile fever, Q fever, avian influenza and Lyme disease, as have been shown in mites from the family Dermanyssidae (Reeves et al. 2006). Despite their ecological relevance, most aspects of the basic biology, ecology and evolution of these mites are still poorly understood. This lack of knowledge, amongst other reasons, is owing to their being very challenging to study; for example, due to most species having typically low prevalence on their hosts and being difficult to sample (being only possible to collect from dead birds).

The family Rhinonyssidae currently includes about 600 described species arranged in eleven genera (Domrow 1969; Fain 1994; Dimov et al. 2015; Dimov 2018). In Parasitology, parasite host-specificity and prevalence are widely-studied parameters as they are informative of relevant processes, such as parasite degree of specialisation, population dynamics or transmission efficiency (Poulin 2011). Host specificity of rhinonyssid mites has been found to vary from one genus to another by surveys across different geographic areas (e.g. USA, Spicer 1987; Canada, Knee et al. 2008). In particular, some genera have been found to be constrained to a single host family, while others can inhabit hosts from different orders (Pence 1975; Butenko 1984). In addition, these studies have found that the prevalence of these mites varies across geographic areas (although the estimates may be biased by a low sample size; Spicer 1987). However, the host-specificity and prevalence of most rhinonyssid mites are still poorly understood, thus hampering further studies on the ecology and evolution of this host-parasite system.

In this study, 2,107 bird specimens from northwest of Russia, belonging to 75 species from 55 genera, 30 families and 10 orders were examined for rhinonyssid mites. The mites were identified and the prevalence (including confidence intervals to show how accurate the estimates are) of these species was calculated. Additionally, the mite-host associations, found in our study, were compared with the known rhinonyssids from these species of birds in the European part of Russia and Europe. Overall, this study represents the largest survey to date carried out on rhinonyssid mites in Russia and one of the most comprehensive datasets on rhinonyssid host-range and prevalence.

## General description

### Purpose

In this survey, 2,107 individual birds were collected in Russia representing 75 species belonging to 55 genera, 30 families and 10 orders and analysed for rhinonyssid mites. The mites were identified and the prevalence (including 95% confidence intervals to show how accurate the estimates are) of these species was calculated. Additionally, the mite-host associations found in our study were compared with the known rhinonyssids from these species of birds in the European part of Russia and Europe.

## Sampling methods

### Study extent

Individual birds were collected during four years (2010-2013).

### Sampling description

Mites were collected from birds that died under various circumstances. Specifically, most of the surveyed hosts were birds found dead on the roads or that died because of high-voltage transmission lines. Host birds were morphologically classified according to Malchevsky and Pukinsky 1983. All the birds were examined for rhinonyssid mites and when they were found, a complete morphometrical study was conducted to identify each specimen (Butenko 1984; Pence 1975; Fain 1956; Dimov and de Rojas 2012; Dimov and Knee 2012; Dimov and Mironov 2012).

### Quality control

The nasal cavity of birds was opened following Butenko's method (Butenko 1984), with some changes introduced by us. In particular, 1) the nasal cavity was opened with a scalpel and scissors under a binocular stereomicroscope. 2) The ossa mandibulae were removed along with the hyoid brush apparatus (apparatus hyobranchialis); the eyeballs were removed with tweezers. 3) Then, two incisions were made: a transverse incision in the region of the papillae pharyngeales and a medial incision, from the rima infundibuli (through the choana) to the ruga palatina mediana area. 4) Nostrils in the area of operculum were examined and then the os maxillare were opened. 5) Lastly, the maxillary bone was removed and the nasal cavity with three conchs was opened, including the largest of all rostral - concha nasalis rostralis, the middle - concha nasalis media and the caudal - concha nasalis caudalis. The examination of all nasal cavities was performed with tweezers and a dissecting needle under a binocular stereomicroscope. Rhinonyssid mites were placed in tubes with 70% ethanol for storage. Each tube was labelled with data on the type of host and a detailed description of the collection site. Mites were then cleared in lactic acid and mounted on slides with Fora-Berlese liquid, according to the generally-accepted technique for small mites (Walter and Krantz 2009; Krantz and Walter 2009). Finally, individual mites were identified, based on morphometrics analysis.

## Geographic coverage

### Description

Individual birds were collected across the northwest of the European part of Russia, mainly in the territory of the Leningrad Region, at 41 points and, to a lesser extent, in territories of Arkhangelsk, Kaliningrad and Pskov regions (Suppl. material 1; Table 2).

### Coordinates

 and 60.05 Latitude; and 31.75 Longitude.

## Taxonomic coverage

### Description

A total of 2,107 individual birds were collected, representing 75 species belonging to 55 genera, 30 families and 10 orders. The majority of the studied hosts (59 species from 36 genera) belonged to the order Passeriformes (the most numerous and widely-distributed order of birds of the northwest of Russia) (Table 1). Specifically, sampled passerine species comprise up to 58% out of the total number of species of passerine species inhabiting the territory of the northwest of Russia (Malchevsky and Pukinsky 1983). In general, 179 mite specimens belonging to 27 species and 8 genera were identified. From a total of 27 host-mite associations, we report 18 novel host-mite associations (Table 2). As expected, due to the higher sampling effort in Passerifoms, the number of new host-mite associations was the highest in this order (9 out of 18), followed by waterbirds belonging to Charadriiformes and Anseriformes (with 4 and 3 new host-mite associations, respectively). Only a single new host-mite association was detected in Caprimulgiformes and Cuculiformes. Lastly, no new association was found in Columbiformes, Galliformes or Piciformes.

### Taxa included

**Table:** 

Rank	Scientific Name	Common Name
kingdom	Animalia	Animals
subkingdom	Eumetazoa	
phylum	Chordata	
subphylum	Vertebrata	
class	Aves	Birds
subclass	Galloanserae	
subclass	Passerae	
superorder	Anserimorphae	
superorder	Columbimorphae	
superorder	Cuculimorphae	
superorder	Gallomorphae	
superorder	Passerimorphae	
order	Accipitriformes	
order	Anseriformes	
order	Caprimulgiformes	
order	Charadriiformes	
order	Columbiformes	
order	Cuculiformes	
order	Galliformes	
order	Gruiformes	
order	Passeriformes	
suborder	Accipitri	
suborder	Anseri	
suborder	Caprimulgi	
suborder	Charadrii	
suborder	Columbi	
suborder	Cuculi	
suborder	Passeri	
suborder	Phasiani	
suborder	Ralli	
superfamily	Accipitroidea	
superfamily	Aegithaloidea	
superfamily	Alaudoidea	
superfamily	Anatoidea	
superfamily	Bombycilloidea	
superfamily	Caprimulgoidea	
superfamily	Charadrioidea	
superfamily	Columboidea	
superfamily	Corvoidea	
superfamily	Cuculoidea	
superfamily	Fringilloidea	
superfamily	Gruoidea	
superfamily	Hirundinoidea	
superfamily	Laroidea	
superfamily	Muscicapoidea	
superfamily	Passeroidea	
superfamily	Phasianoidea	
superfamily	Ralloidea	
superfamily	Reguloidea	
superfamily	Scolopacoidea	
superfamily	Sittoidea	
superfamily	Sturnoidea	
superfamily	Sylvioidea	
family	Accipitridae	
family	Anatidae	
family	Caprimulgidae	
family	Charadriidae	
family	Columbidae	
family	Corvidae	
family	Cuculidae	
family	Emberizidae	
family	Fringillidae	
family	Laridae	
family	Motacillidae	
family	Muscicapidae	
family	Paridae	
family	Ploceidae	
family	Rallidae	
family	Regulidae	
family	Scolopacidae	
family	Sturnidae	
family	Sylviidae	
family	Turdidae	
species	*Acanthis canabina*	
species	*Acanthis flammea*	
species	*Aegithalos caudatus*	
species	*Accipiter nisus*	
species	*Alauda arvensis*	
species	*Anas crecca*	
species	*Anas platyrhynchos*	
species	*Anthus pratensis*	
species	*Anthus trivialis*	
species	*Aythya nyroca*	
species	*Bombycilla garrulus*	
species	*Buteo buteo*	
species	*Caprimulgus europeus*	
species	*Carduelis carduelis*	
species	*Carpodacus erythrinus*	
species	*Charadrius dubius*	
species	*Chloris chloris*	
species	*Columba livia*	
species	*Corvus cornix*	
species	*Coturnix coturnix*	
species	*Cuculus canorus*	
species	*Delichon urbica*	
species	*Dendrocopos major*	
species	*Emberiza citrinella*	
species	*Emberiza hortulana*	
species	*Emberiza rustica*	
species	*Emberiza schoeniclus*	
species	*Ficedula hypoleuca*	
species	*Fringilla coelebs*	
species	*Fringilla montifringilla*	
species	*Fulica atra*	
species	*Gallinula chloropus*	
species	*Garrulus glandarius*	
species	*Hirundo rustica*	
species	*Larus ridibundus*	
species	*Loxia curvirostra*	
species	*Luscinia luscinia*	
species	*Motacilla alba*	
species	*Motacilla flava*	
species	*Muscicapa striata*	
species	*Oenanthe oenanthe*	
species	*Parus ater*	
species	*Parus caeruleus*	
species	*Parus cristatus*	
species	*Parus major*	
species	*Parus montanus*	
species	*Parus palustris*	
species	*Passer domesticus*	
species	*Passer montanus*	
species	*Phylloscopus sibilatrix*	
species	*Phylloscopus trochilus*	
species	*Pica pica*	
species	*Pinicola enucleator*	
species	*Pyrrhula pyrrhula*	
species	*Regulus regulus*	
species	*Riparia riparia*	
species	*Saxicola rubetra*	
species	*Scolopax rusticola*	
species	*Serinus canaria*	
species	*Serinus serinus*	
species	*Sitta europea*	
species	*Somateria mollissima*	
species	*Spinus spinus*	
species	*Sterna hirundo*	
species	*Sturnus vulgaris*	
species	*Sylvia atricapilla*	
species	*Sylvia borin*	
species	*Sylvia curruca*	
species	*Turdus iliacus*	
species	*Turdus merula*	
species	*Turdus philomelos*	
species	*Turdus pilaris*	
species	*Vanellus vanellus*	
kingdom	Animalia	Animals
subkingdom	Eumetazoa	
phylum	Chordata	
phylum	Arthropoda	Arthropods
subphylum	Vertebrata	
subphylum	Chelicerata	
class	Aves	Birds
class	Arachnida	
subclass	Galloanserae	
subclass	Passerae	
subclass	Acari	Mites
superorder	Anserimorphae	
superorder	Columbimorphae	
superorder	Cuculimorphae	
superorder	Gallomorphae	
superorder	Passerimorphae	
superorder	Parasitiformes	
order	Accipitriformes	
order	Anseriformes	
order	Caprimulgiformes	
order	Charadriiformes	
order	Columbiformes	
order	Cuculiformes	
order	Galliformes	
order	Gruiformes	
order	Passeriformes	
order	Mesostigmata	
suborder	Accipitri	
suborder	Anseri	
suborder	Caprimulgi	
suborder	Charadrii	
suborder	Columbi	
suborder	Cuculi	
suborder	Passeri	
suborder	Phasiani	
suborder	Ralli	
suborder	Monogynaspida	
infraorder	Gamasina	
superfamily	Accipitroidea	
superfamily	Aegithaloidea	
superfamily	Alaudoidea	
superfamily	Anatoidea	
superfamily	Bombycilloidea	
superfamily	Caprimulgoidea	
superfamily	Charadrioidea	
superfamily	Columboidea	
superfamily	Corvoidea	
superfamily	Cuculoidea	
superfamily	Fringilloidea	
superfamily	Gruoidea	
superfamily	Hirundinoidea	
superfamily	Laroidea	
superfamily	Muscicapoidea	
superfamily	Passeroidea	
superfamily	Phasianoidea	
superfamily	Ralloidea	
superfamily	Reguloidea	
superfamily	Scolopacoidea	
superfamily	Sittoidea	
superfamily	Sturnoidea	
superfamily	Sylvioidea	
superfamily	Dermanyssoidea	
family	Accipitridae	
family	Anatidae	
family	Caprimulgidae	
family	Charadriidae	
family	Columbidae	
family	Corvidae	
family	Cuculidae	
family	Emberizidae	
family	Fringillidae	
family	Laridae	
family	Motacillidae	
family	Muscicapidae	
family	Paridae	
family	Ploceidae	
family	Rallidae	
family	Regulidae	
family	Scolopacidae	
family	Sturnidae	
family	Sylviidae	
family	Turdidae	
family	Rhinonyssidae	Rhinonyssids
species	*Larinyssus iohanssenae*	
species	*Larinyssus orbicularis*	
species	*Mesonyssus columbae*	
species	*Mesonyssus melloi*	
species	*Ptilonyssus degtiarevae*	
species	*Ptilonyssus euroturdi*	
species	*Ptilonyssus hirsti*	
species	*Ptilonyssus lovottiae*	
species	*Ptilonyssus mironovi*	
species	*Ptilonyssus motacillae*	
species	*Ptilonyssus pari*	
species	*Ptilonyssus sairae*	
species	*Ptilonyssus schumili*	
species	*Rallinyssus caudistigmus*	
species	*Rhinonyssus bregetovae*	
species	*Rhinonyssus dobromiri*	
species	*Rhinonyssus kadrae*	
species	*Rhinonyssus levinsini*	
species	*Rhinonyssus neglectus*	
species	*Rhinonyssus nyrocae*	
species	*Rhinonyssus polystictae*	
species	*Rhinonyssus subrhinolethrum*	
species	*Sternostoma dureni*	
species	*Sternostoma marchae*	
species	*Sternostoma turdi*	
species	*Sternostoma zini*	
species	*Vitznyssus tsachevi*	

## Usage rights

### Use license

Creative Commons Public Domain Waiver (CC-Zero)

## Data resources

### Data package title

Host-mite associations of rhinonyssid mites (Mesostigmata: Rhinonyssidae) in Northwest Russia.

### Number of data sets

1

### Data set 1.

#### Data set name

Table S2

#### Number of columns

5

#### Description

Host-mite associations form a total of 2,107 individual hosts totalling 75 bird species from 30 avian families and 10 orders (Suppl. material 3). Information on eight mite genera and 28 mite species from Rhinonyssidae is presented. Each row depicts an individual bird from mite-host associations, in which more than one mite was found. See Table 2 for more information (e.g. prevalence).

**Data set 1.** 

Column label	Column description
Bird taxa	Scientific name and authority of bird taxa
Mite species	Scientific name and authority of mite taxa
Locality (this study)	Sampling localities
Coordinates	Coordinates in degrees minutes and seconds
Date	Collection date

## Additional information

### Discussion

In the present study, the rhinonyssid mite species collected from 2,107 bird individuals from 75 bird species at 41 sites in Northwest Russia were reported. A total of 27 host-mite associations were found, from which 18 were novel (Table 2).

The prevalence of rhinonyssid mites was found to vary between bird taxa, as found by previous studies (Spicer 1987; Table 1). Interestingly, the prevalence values found here were lower overall (particularly low in Passeriformes) than those found by previous studies (e.g. 4.41%, this study vs. 17%, USA, Spicer 1987; or 15-16%, Canada, Knee 2018). The lower values found here may be due to differences in climatic conditions, as have been found by previous studies (e.g. Spicer 1987). Overall, our results support current expectations that rhinonyssid mites are generally associated with low prevalence with their hosts (Spicer 1987;Knee 2018).

The mite-host associations found in this study were compared with those known from the same host species inhabiting the European part of Russia and Europe (see Suppl. material 2). In particular, a higher number of mite species of rhinonyssid genera was found in the northwest of Russia compared to the European part of Russia and Western Europe (Suppl. material 2). This pattern was particularly noticeable for species-rich genera, such as *Sternostoma, Mesonyssus, Rhinonyssus* and *Ptilonyssus*. In addition, the pattern was most acute between the northwest of Russia and western Europe. For instance, there were almost no common species between these two areas (only 14 common species from six genera). In contrast, almost all genera (five out of eight) of Rhinonyssidae were shared.

On the other hand, some species that have been found in the European part of Russia and Europe were not found in this study (e.g. *Mesonyssus hirsutus* from *Columba livia; Ptilonyssus nudus* from *Fringilla coelebs; Ptilonyssus pari* from *Parus ater; Parus caeruleus or Paruseuropea; Rhinonyssusvanellus* from *Vanellus vanellus*). Overall, these differences in diversity could be the outcome of the lack of knowledge about these mites in these regions. Indeed, differences in sampling effort (i.e. some groups have been more extensively sampled in Northwest Russia than in other geographic areas) may be biasing these interpretations. Overall, further studies aimed at ascertaining whether specific rhinonyssid mite species are found throughout all their host distribution are encouraged. In this vein, global syntheses are needed to draw more general conclusions on the distribution of rhinonyssid mites. Additionally, future studies describing new species are required to catalogue the unknown diversity of this group of mites. In addition, new molecular approaches (e.g. DNA metabarcoding of complex samples; Doña et al. 2019) would help to accelerate the discovery of new species along with validating the species status of previously-described rhinonyssid species, as cryptic species are known in this group (de Rojas et al. 2018).

## Supplementary Material

523D0A13-6045-50C5-B2EF-66E3C824F0EF 10.3897/BDJ.8.e49535.suppl1 Supplementary material 1 Figure S1 Data type: Figure
Brief description: Map of sampling localities. Note that specific locations, as well as their coordinates, can be found in Table 2.
File: oo_365465.png https://binary.pensoft.net/file/365465 de Rojas M, Doña J, Dimov I

2FD7E6A9-09C9-5067-99BC-C40C1094983F 10.3897/BDJ.8.e49535.suppl2 Supplementary material 2 Table S1 Data type: Table
Brief description: Comparison of species of the family Rhinonyssidae located in Northwest Russia, the European part of Russia and Western Europe; plus and minus signs indicate presence and absence, respectively. Mite species found in this study are marked with *
File: oo_365467.tsv https://binary.pensoft.net/file/365467 de Rojas M, Doña J, Dimov I

615F5B64-5FE2-59E5-BD87-A9C012E357EE 10.3897/BDJ.8.e49535.suppl3 Supplementary material 3 Table S2 Data type: Table
Brief description: Rhinonyssid mites from Russian birds
File: oo_365526.tsv https://binary.pensoft.net/file/365526 de Rojas, M., Doña, J., Dimov, I

## Figures and Tables

**Table 1.** Data of prevalence of rhinonyssid mites in different orders of hosts and the number of families, genera and species of birds studied. Confidence intervals of prevalence (95%) are provided between parentheses.

**Bird order**	**Bird families**	**Bird genera**	**Bird species**	**Analysed individuals**	**Infected individuals**	**Prevalence**
Anseriformes	1	3	4	105	13	12.38% (7.38-20.04)
Caprimulgiformes	1	1	1	2	1	50% (2.56-97.44)
Charadriiformes	4	5	6	117	8	6.8% (3.51-12.91)
Columbiformes	1	1	1	262	10	3.8% (2.09-6.88)
Cuculiformes	1	1	1	13	1	7.69% (0.39-33.31)
Accipitriformes	1	2	2	3	0	0% (0-56.15)
Galliformes	1	2	2	32	0	0% (0-10.71)
Gruiformes	1	2	2	8	2	25% (4.44-59.07)
Passeriformes	18	37	55	1549	58	3.7% (2.91-4.81)
Piciformes	1	1	1	16	0	0% (0-19.36)
**Total**	**30**	**55**	**75**	**2107**	**93**	**4.41% (3.62-5.38)**

**Table 2.** Data on the mite-host associations detected. Each row depicts an individual bird from mite-host associations, in which more than one mite was found. N = number of birds examined per mite-host association, Ni = Number of infected birds, I = number of mites isolated. Region (previous records) = NR: Northwest Russia, ER: European part of Russia, WE: Western Europe and * indicates a previously unrecorded mite host association. Note that values from N and Ni columns are duplicated between individual birds that belong to the same mite-host association.

**Bird taxa**	**Mite species**	**N**	**Ni**	**I**	**Region (previous records)**	**Locality (this study)**	**Previous records**
*Accipiter nisus* (Linnaeus, 1758)	NA	2	0	0	NA	NA	NA
*Buteo buteo* (Linnaeus, 1758)	NA	1	0	0	NA	NA	NA
*Anas platyrhynchos* Linnaeus, 1758	*Rhinonyssus kadrae* Dimov, 2013	38	1	2	NR*	Dubrovka, Leningrad Oblast, Russia	NA
*Anas platyrhynchos* Linnaeus, 1758	*Rhinonyssus levinseni* (Tragardh, 1904)	38	1	1	NR*	Volosovo, Leningrad Region, Russia	NA
*Aythya nyroca* (Güldenstädt, 1770)	*Rhinonyssus nyrocae* Butenko, 1971	1	1	1	NR, WE	Pskov, Pskov region, Russia	Butenko 1971, Butenko 1984
*Somateria mollissima* Linnaeus, 1758	*Rhinonyssus polystictae* Butenko, 1984	11	9	1	NR*	Ermilovo, Leningrad Region, Russia	NA
*Somateria mollissima* Linnaeus, 1758	*Rhinonyssus polystictae* Butenko, 1984	11	9	1	NR*	Ermilovo, Leningrad Region, Russia	NA
*Somateria mollissima* Linnaeus, 1758	*Rhinonyssus polystictae* Butenko, 1984	11	9	2	NR*	Ermilovo, Leningrad Region, Russia	NA
*Somateria mollissima* Linnaeus, 1758	*Rhinonyssus polystictae* Butenko, 1984	11	9	1	NR*	Ermilovo, Leningrad Region, Russia	NA
*Somateria mollissima* Linnaeus, 1758	*Rhinonyssus polystictae* Butenko, 1984	11	9	2	NR*	Ermilovo, Leningrad Region, Russia	NA
*Somateria mollissima* Linnaeus, 1758	*Rhinonyssus polystictae* Butenko, 1984	11	9	1	NR*	Ermilovo, Leningrad Region, Russia	NA
*Somateria mollissima* Linnaeus, 1758	*Rhinonyssus polystictae* Butenko, 1984	11	9	2	NR*	Ermilovo, Leningrad Region, Russia	NA
*Somateria mollissima* Linnaeus, 1758	*Rhinonyssus polystictae* Butenko, 1984	11	9	1	NR*	Ermilovo, Leningrad Region, Russia	NA
*Somateria mollissima* Linnaeus, 1758	*Rhinonyssus polystictae* Butenko, 1984	11	9	1	NR*	Ermilovo, Leningrad Region, Russia	NA
*Anas crecca* Linnaeus, 1758	*Rhinonyssus subrhinolethrum* Butenko, 1971	17	1	1	NR	Pikalevo, Leningrad Region, Russia	Butenko 1971, Butenko 1984
*Caprimulgus europeus* Linnaeus, 1758	*Vitznyssus tsachevi* Dimov et Rojas 2012	2	1	2	NR*	Pikalevo, Leningrad region, Russia	NA
*Charadrius dubius* Scopoli, 1786	*Rhinonyssus bregetovae* Butenko, 1974	27	3	4	NR	Voypala, Leningrad Region, Russia	Butenko 1984
*Charadrius dubius* Scopoli, 1786	*Rhinonyssus bregetovae* Butenko, 1974	27	3	2	NR	Voypala, Leningrad Region, Russia	Butenko 1984
*Charadrius dubius* Scopoli, 1786	*Rhinonyssus bregetovae* Butenko, 1974	27	3	1	NR	Voypala, Leningrad Region, Russia	Butenko 1984
*Charadrius dubius* Scopoli, 1786	*Rhinonyssus neglectus* Hirst 1921	27	1	1	NR*	Lavrovo, Leningrad Region, Russia	NA
*Vanellus vanellus* Linnaeus, 1758	*Rhinonyssus dobromiri* Dimov et Spicer, 2013	4	1	2	NR*	Leningrad Region, Russia	NA
*Larus argentatus* Pontoppidan, 1763	*Larinyssus orbicularis* Strandtmann, 1948	22	1	2	NR*	Voybokalo, Leningrad Oblast, Russia	NA
*Larus ridibundus* Linnaeus, 1766	NA	19	0	0	NA	NA	NA
*Scolopax rusticola* Linnaeus, 1758	NA	1	0	0	NA	NA	NA
*Sterna hirundo* Linnaeus, 1758	*Larinyssus iohanssenae* Dimov, 2013	17	2	1	NR*	Kronstad, Leningrad Region, Russia; Apraksin, Leningrad Region, Russia	NA
*Sterna hirundo* Linnaeus, 1758	*Larinyssus iohanssenae* Dimov, 2013	17	2	2	NR*	Kronstad, Leningrad Region, Russia; Apraksin, Leningrad Region, Russia	NA
*Columba livia* Gmelin, 1789	*Mesonyssus columbae* Crossley, 1950	262	5	3	NR, WE	St. Petersburg, Russia; Voybokalo, Leningrad Oblast, Russia; Slantsy, Leningrad Region, Russia; Luga, Leningradskaya, Russia; Radogosh, Leningrad Region, Russia	Butenko 1984; Cerny 1970; Crossley 1951; Domrow 1965; Domrow 1966a; Domrow 1966b; Fain 1956; Fain 1957; Fain 1958; Fain 1962b; Fain et al. 1974; Pence 1975; Sixl 1971; Wilson 1964; Wilson 1966; Zumpt and Till 1955
*Columba livia* Gmelin, 1789	*Mesonyssus columbae* Crossley, 1950	262	5	1	NR, WE	St. Petersburg, Russia; Voybokalo, Leningrad Oblast, Russia; Slantsy, Leningrad Region, Russia; Luga, Leningradskaya, Russia; Radogosh, Leningrad Region, Russia	Butenko 1984; Cerny 1970; Crossley 1951; Domrow 1965; Domrow 1966a; Domrow 1966b; Fain 1956; Fain 1957; Fain 1958; Fain 1962b; Fain et al. 1974; Pence 1975; Sixl 1971; Wilson 1964; Wilson 1966; Zumpt and Till 1955
*Columba livia* Gmelin, 1789	*Mesonyssus columbae* Crossley, 1950	262	5	2	NR, WE	St. Petersburg, Russia; Voybokalo, Leningrad Oblast, Russia; Slantsy, Leningrad Region, Russia; Luga, Leningradskaya, Russia; Radogosh, Leningrad Region, Russia	Butenko 1984; Cerny 1970; Crossley 1951; Domrow 1965; Domrow 1966a; Domrow 1966b; Fain 1956; Fain 1957; Fain 1958; Fain 1962b; Fain et al. 1974; Pence 1975; Sixl 1971; Wilson 1964; Wilson 1966; Zumpt and Till 1955
*Columba livia* Gmelin, 1789	*Mesonyssus columbae* Crossley, 1950	262	5	2	NR, WE	St. Petersburg, Russia; Voybokalo, Leningrad Oblast, Russia; Slantsy, Leningrad Region, Russia; Luga, Leningradskaya, Russia; Radogosh, Leningrad Region, Russia	Butenko 1984; Cerny 1970; Crossley 1951; Domrow 1965; Domrow 1966a; Domrow 1966b; Fain 1956; Fain 1957; Fain 1958; Fain 1962b; Fain et al. 1974; Pence 1975; Sixl 1971; Wilson 1964; Wilson 1966; Zumpt and Till 1955
*Columba livia* Gmelin, 1789	*Mesonyssus columbae* Crossley, 1950	262	5	3	NR, WE	St. Petersburg, Russia; Voybokalo, Leningrad Oblast, Russia; Slantsy, Leningrad Region, Russia; Luga, Leningradskaya, Russia; Radogosh, Leningrad Region, Russia	Butenko 1984; Cerny 1970; Crossley 1951; Domrow 1965; Domrow 1966a; Domrow 1966b; Fain 1956; Fain 1957; Fain 1958; Fain 1962b; Fain et al. 1974; Pence 1975; Sixl 1971; Wilson 1964; Wilson 1966; Zumpt and Till 1955
*Columba livia* Gmelin, 1789	*Mesonyssus melloi* Castro, 1948	262	3	4	NR, WE	St. Petersburg, Russia; Voybokalo, Leningrad Oblast, Russia; Slantsy, Leningrad Region, Russia.	Butenko 1984; Castro 1948; Domrow 1966a; Domrow 1969; Domrow 1972a; Domrow 1972b; Fain 1959; Fain 1962b; Pence 1979; Sixl 1969; Wilson 1964; Wilson 1966; Zumpt and Till 1955
*Columba livia* Gmelin, 1789	*Mesonyssus melloi* Castro, 1948	262	3	4	NR, WE	St. Petersburg, Russia; Voybokalo, Leningrad Oblast, Russia; Slantsy, Leningrad Region, Russia.	Butenko 1984; Castro 1948; Domrow 1966a; Domrow 1969; Domrow 1972a; Domrow 1972b; Fain 1959; Fain 1962b; Pence 1979; Sixl 1969; Wilson 1964; Wilson 1966; Zumpt and Till 1955
*Columba livia* Gmelin, 1789	*Mesonyssus melloi* Castro, 1948	262	3	2	NR, WE	St. Petersburg, Russia; Voybokalo, Leningrad Oblast, Russia; Slantsy, Leningrad Region, Russia.	Butenko 1984; Castro 1948; Domrow 1966a; Domrow 1969; Domrow 1972a; Domrow 1972b; Fain 1959; Fain 1962b; Pence 1979; Sixl 1969; Wilson 1964; Wilson 1966; Zumpt and Till 1955
*Cuculus canorus* Linnaeus, 1758	*Sternostoma zini* Dimov et Knee, 2012	13	1	2	NR*	Vyritsa, Leningrad Region, Russia	NA
*Coturnix coturnix* Linnaeus, 1758	NA	31	0	0	NA	NA	NA
*Tetrao urogallus* Linnaeus, 1758	NA	1	0	0	NA	NA	NA
*Gallinula chloropus* Linnaeus, 1758	*Rallinyssus caudistigmus* Strandtmann, 1948	6	1	3	NR, WE	Gatchina, Leningrad Region, Russia; Sosnovy Bor, Leningrad Region, Russia	Domrow 1966a; Domrow 1969; Fain 1957; Fain et al. 1974; Pence 1975; Strandtmann 1948
*Fulica atra* Linnaeus, 1758	*Rallinyssus caudistigmus* Strandtmann, 1948	2	1	2	NR, ER, WE	Gatchina, Leningrad Region, Russia; Sosnovy Bor, Leningrad Region, Russia	Bregetova 1951; Butenko 1984; Domrow 1966a; Domrow 1969; Fain 1957; Fain 1959; Fain et al. 1974; Pence 1972d; Pence 1975; Strandtmann 1948
*Alauda arvensis* Linnaeus, 1758	*Ptilonyssus schumili* Butenko et Lavrovskaya, 1980	35	6	2	NR, ER	Rjbachii, Leningrad Region, Russia	Butenko and Lavroskaya 1980a; Butenko and Lavroskaya 1980b
*Alauda arvensis* Linnaeus, 1758	*Ptilonyssus schumili* Butenko et Lavrovskaya, 1980	35	6	1	NR, ER	Rjbachii, Leningrad Region, Russia	Butenko and Lavroskaya 1980a; Butenko and Lavroskaya 1980b
*Alauda arvensis* Linnaeus, 1758	*Ptilonyssus schumili* Butenko et Lavrovskaya, 1980	35	6	2	NR, ER	Rjbachii, Leningrad Region, Russia	Butenko and Lavroskaya 1980a; Butenko and Lavroskaya 1980b
*Alauda arvensis* Linnaeus, 1758	*Ptilonyssus schumili* Butenko et Lavrovskaya, 1980	35	6	1	NR, ER	Rjbachii, Leningrad Region, Russia	Butenko and Lavroskaya 1980a; Butenko and Lavroskaya 1980b
*Alauda arvensis* Linnaeus, 1758	*Ptilonyssus schumili* Butenko et Lavrovskaya, 1980	35	6	1	NR, ER	Rjbachii, Leningrad Region, Russia	Butenko and Lavroskaya 1980a; Butenko and Lavroskaya 1980b
*Alauda arvensis* Linnaeus, 1758	*Ptilonyssus schumili* Butenko et Lavrovskaya, 1980	35	6	2	NR, ER	Rjbachii, Leningrad Region, Russia	Butenko and Lavroskaya 1980a; Butenko and Lavroskaya 1980b
*Aegithalos caudatus* Linnaeus, 1758	NA	9	0	0	NA	NA	NA
*Bombycilla garrulus* Linnaeus, 1758	NA	1	0	0	NA	NA	NA
*Corvus cornix* Linnaeus, 1758	NA	38	0	0	NA	NA	NA
*Garrulus glandarius* Linnaeus, 1758	NA	3	0	0	NA	NA	NA
*Pica pica* Linnaeus, 1758	NA	32	0	0	NA	NA	NA
*Emberiza citrinella* Linnaeus, 1758	NA	29	0	0	NA	NA	NA
*Emberiza hortulana* Linnaeus, 1758	NA	1	0	0	NA	NA	NA
*Emberiza rustica* (Pallas, 1776)	NA	5	0	0	NA	NA	NA
*Emberiza schoeniclus* Linnaeus, 1758	NA	2	0	0	NA	NA	NA
*Acanthis canabina* Linnaeus, 1758	NA	28	0	0	NA	NA	NA
*Acanthis flammea* Linnaeus, 1758	NA	7	0	0	NA	NA	NA
*Fringilla coelebs* Linnaeus, 1758	*Ptilonyssus hirsti* (Castro et Pereira, 1947)	71	11	1	NR*	Grjazno, Kaliningrado Region, Russia	NA
*Fringilla coelebs* Linnaeus, 1758	*Ptilonyssus hirsti* (Castro et Pereira, 1947)	71	11	2	NR*	Grjazno, Kaliningrado Region, Russia	NA
*Fringilla coelebs* Linnaeus, 1758	*Ptilonyssus hirsti* (Castro et Pereira, 1947)	71	11	2	NR*	Grjazno, Kaliningrado Region, Russia	NA
*Fringilla coelebs* Linnaeus, 1758	*Ptilonyssus hirsti* (Castro et Pereira, 1947)	71	11	1	NR*	Grjazno, Kaliningrado Region, Russia	NA
*Fringilla coelebs* Linnaeus, 1758	*Ptilonyssus hirsti* (Castro et Pereira, 1947)	71	11	1	NR*	Grjazno, Kaliningrado Region, Russia	NA
*Fringilla coelebs* Linnaeus, 1758	*Ptilonyssus hirsti* (Castro et Pereira, 1947)	71	11	3	NR*	Grjazno, Kaliningrado Region, Russia	NA
*Fringilla coelebs* Linnaeus, 1758	*Ptilonyssus hirsti* (Castro et Pereira, 1947)	71	11	1	NR*	Grjazno, Kaliningrado Region, Russia	NA
*Fringilla coelebs* Linnaeus, 1758	*Ptilonyssus hirsti* (Castro et Pereira, 1947)	71	11	1	NR*	Grjazno, Kaliningrado Region, Russia	NA
*Fringilla coelebs* Linnaeus, 1758	*Ptilonyssus hirsti* (Castro et Pereira, 1947)	71	11	3	NR*	Grjazno, Kaliningrado Region, Russia	NA
*Fringilla coelebs* Linnaeus, 1758	*Ptilonyssus hirsti* (Castro et Pereira, 1947)	71	11	2	NR*	Grjazno, Kaliningrado Region, Russia	NA
*Fringilla coelebs* Linnaeus, 1758	*Ptilonyssus hirsti* (Castro et Pereira, 1947)	71	11	1	NR*	Grjazno, Kaliningrado Region, Russia	NA
*Carduelis carduelis* Linnaeus, 1758	NA	39	0	0	NA	NA	NA
*Carpodacus erythrinus* (Pallas, 1770)	NA	3	0	0	NA	NA	NA
*Chloris chloris* Linnaeus, 1758	NA	31	0	0	NA	NA	NA
*Fringilla montifringilla* Linnaeus, 1758	NA	1	0	0	NA	NA	NA
*Loxia curvirostra* Linnaeus, 1758	NA	51	0	0	NA	NA	NA
*Pinicola enucleator* Linnaeus, 1758	NA	16	0	0	NA	NA	NA
*Pyrrhula pyrrhula* Linnaeus, 1758	NA	9	0	0	NA	NA	NA
*Serinus serinus* Linnaeus, 1766	NA	5	0	0	NA	NA	NA
*Serinus canaria* Linnaeus, 1758	*Sternostoma marchae* Dimov, 2012	29	1	10	NR*	St. Petersburg, Russia	NA
*Spinus spinus* Linnaeus, 1758	NA	13	0	0	NA	NA	NA
*Delichon urbica* Linnaeus, 1758	NA	47	0	0	NA	NA	NA
*Hirundo rustica* Linnaeus, 1758	NA	63	0	0	NA	NA	NA
*Riparia riparia* Linnaeus, 1758	NA	23	0	0	NA	NA	NA
*Anthus pratensis* Linnaeus, 1758	NA	1	0	0	NA	NA	NA
*Anthus trivialis* Linnaeus, 1758	NA	17	0	0	NA	NA	NA
*Motacilla flava* Linnaeus, 1758	NA	7	0	0	NA	NA	NA
*Motacilla alba* Linnaeus, 1758	NA	86	0	0	NA	NA	NA
*Oenanthe oenanthe* Linnaeus, 1758	*Ptilonyssus motacillae* Fain, 1956	91	3	3	NR*	Tikhvin, Leningrad Oblast, Russia; Sinyavino, Leningrad Oblast, Russia	NA
*Oenanthe oenanthe* Linnaeus, 1758	*Ptilonyssus motacillae* Fain, 1956	91	3	2	NR*	Tikhvin, Leningrad Oblast, Russia; Sinyavino, Leningrad Oblast, Russia	NA
*Oenanthe oenanthe* Linnaeus, 1758	*Ptilonyssus motacillae* Fain, 1956	91	3	3	NR*	Tikhvin, Leningrad Oblast, Russia; Sinyavino, Leningrad Oblast, Russia	NA
*Ficedula hypoleuca* (Pallas, 1764)	NA	2	0	0	NA	NA	NA
*Luscinia luscinia* Linnaeus, 1758	NA	8	0	0	NA	NA	NA
*Muscicapa striata* (Pallas, 1764)	NA	27	0	0	NA	NA	NA
*Saxicola rubetra* Linnaeus, 1758	NA	1	0	0	NA	NA	NA
*Parus caeruleus* Linnaeus, 1758	*Ptilonyssus mironovi* Dimov, 2012	19	1	3	NR*	Lomonosov, Leningrad Region, Russia	NA
*Parus major* Linnaeus, 1758	*Ptilonyssus sairae* Castro, 1948	118	3	2	NR*	St. Petersburg, Russia	NA
*Parus major* Linnaeus, 1758	*Ptilonyssus sairae* Castro, 1948	118	3	2	NR*	St. Petersburg, Russia	NA
*Parus major* Linnaeus, 1758	*Ptilonyssus sairae* Castro, 1948	118	3	1	NR*	St. Petersburg, Russia	NA
*Parus major* Linnaeus, 1758	*Ptilonyssus pari* Fain et Hyland 1963	118	9	2	NR, WE	St. Petersburg, Russia	Fain and Bafort 1963; Fain and Hyland 1963; Fain et al. 1974; Kadosaka et al. 1983; Pence 1972a; Pence 1972b; Pence 1975; Pence and Casto 1976; Shumilo and Lunkashu 1970; Sixl 1969; Sixl 1970; Spicer 1977; Spicer 1978
*Parus major* Linnaeus, 1758	*Ptilonyssus pari* Fain et Hyland 1963	118	9	1	NR, WE	St. Petersburg, Russia	Fain and Bafort 1963; Fain and Hyland 1963; Fain et al. 1974; Kadosaka et al. 1983; Pence 1972a; Pence 1972b; Pence 1975; Pence and Casto 1976; Shumilo and Lunkashu 1970; Sixl 1969; Sixl 1970; Spicer 1977; Spicer 1978
*Parus major* Linnaeus, 1758	*Ptilonyssus pari* Fain et Hyland 1963	118	9	1	NR, WE	St. Petersburg, Russia	Fain and Bafort 1963; Fain and Hyland 1963; Fain et al. 1974; Kadosaka et al. 1983; Pence 1972a; Pence 1972b; Pence 1975; Pence and Casto 1976; Shumilo and Lunkashu 1970; Sixl 1969; Sixl 1970; Spicer 1977; Spicer 1978
*Parus major* Linnaeus, 1758	*Ptilonyssus pari* Fain et Hyland 1963	118	9	1	NR, WE	St. Petersburg, Russia	Fain and Bafort 1963; Fain and Hyland 1963; Fain et al. 1974; Kadosaka et al. 1983; Pence 1972a; Pence 1972b; Pence 1975; Pence and Casto 1976; Shumilo and Lunkashu 1970; Sixl 1969; Sixl 1970; Spicer 1977; Spicer 1978
*Parus major* Linnaeus, 1758	*Ptilonyssus pari* Fain et Hyland 1963	118	9	1	NR, WE	St. Petersburg, Russia	Fain and Bafort 1963; Fain and Hyland 1963; Fain et al. 1974; Kadosaka et al. 1983; Pence 1972a; Pence 1972b; Pence 1975; Pence and Casto 1976; Shumilo and Lunkashu 1970; Sixl 1969; Sixl 1970; Spicer 1977; Spicer 1978
*Parus major* Linnaeus, 1758	*Ptilonyssus pari* Fain et Hyland 1963	118	9	1	NR, WE	St. Petersburg, Russia	Fain and Bafort 1963; Fain and Hyland 1963; Fain et al. 1974; Kadosaka et al. 1983; Pence 1972a; Pence 1972b; Pence 1975; Pence and Casto 1976; Shumilo and Lunkashu 1970; Sixl 1969; Sixl 1970; Spicer 1977; Spicer 1978
*Parus major* Linnaeus, 1758	*Ptilonyssus pari* Fain et Hyland 1963	118	9	1	NR, WE	St. Petersburg, Russia	Fain and Bafort 1963; Fain and Hyland 1963; Fain et al. 1974; Kadosaka et al. 1983; Pence 1972a; Pence 1972b; Pence 1975; Pence and Casto 1976; Shumilo and Lunkashu 1970; Sixl 1969; Sixl 1970; Spicer 1977; Spicer 1978
*Parus major* Linnaeus, 1758	*Ptilonyssus pari* Fain et Hyland 1963	118	9	2	NR, WE	St. Petersburg, Russia	Fain and Bafort 1963; Fain and Hyland 1963; Fain et al. 1974; Kadosaka et al. 1983; Pence 1972a; Pence 1972b; Pence 1975; Pence and Casto 1976; Shumilo and Lunkashu 1970; Sixl 1969; Sixl 1970; Spicer 1977; Spicer 1978
*Parus major* Linnaeus, 1758	*Ptilonyssus pari* Fain et Hyland 1963	118	9	1	NR, WE	St. Petersburg, Russia	Fain and Bafort 1963; Fain and Hyland 1963; Fain et al. 1974; Kadosaka et al. 1983; Pence 1972a; Pence 1972b; Pence 1975; Pence and Casto 1976; Shumilo and Lunkashu 1970; Sixl 1969; Sixl 1970; Spicer 1977; Spicer 1978
*Parus ater* Linnaeus, 1758	NA	2	0	0	NA	NA	NA
*Parus cristatus* Linnaeus, 1758	NA	9	0	0	NA	NA	NA
*Parus montanus* (Conrad von Baldenstein, 1827)	NA	29	0	0	NA	NA	NA
*Parus palustris* Linnaeus, 1758	NA	6	0	0	NA	NA	NA
*Passer domesticus* Linnaeus, 1758	*Ptilonyssus degtiarevae* Dimov et Mironov, 2012	74	1	5	NR*	Gavrilovo, Leningradskaya Region, Russia	NA
*Passer montanus* Linnaeus, 1758	*Ptilonyssus lovottiae* Dimov et Mironov, 2012	91	3	18	NR*	Leningrad Region Russia; Boronichevo, Leningrad Region, Russia; Novaya LadogaLeningrad Oblast, Russia	NA
*Passer montanus* Linnaeus, 1758	*Ptilonyssus lovottiae* Dimov et Mironov, 2012	91	3	3	NR*	Leningrad Region Russia; Boronichevo, Leningrad Region, Russia; Novaya LadogaLeningrad Oblast, Russia	NA
*Passer montanus* Linnaeus, 1758	*Ptilonyssus lovottiae* Dimov et Mironov, 2012	91	3	5	NR*	Leningrad Region Russia; Boronichevo, Leningrad Region, Russia; Novaya LadogaLeningrad Oblast, Russia	NA
*Regulus regulus* Linnaeus, 1758	NA	27	0	0	NA	NA	NA
*Sitta europea* Linnaeus, 1758	NA	3	0	0	NA	NA	NA
*Phylloscopus sibilatrix* (Bechstein, 1793)	NA	1	0	0	NA	NA	NA
*Phylloscopus trochilus* Linnaeus, 1758	NA	16	0	0	NA	NA	NA
*Dendrocopos major* Linnaeus, 1758	NA	16	0	0	NA	NA	NA
*Sturnus vulgaris* Linnaeus, 1758	NA	39	0	0	NA	NA	NA
*Sylvia atricapilla* Linnaeus, 1758	NA	7	0	0	NA	NA	NA
*Sylvia borin* (Boddaert, 1783)	NA	3	0	0	NA	NA	NA
*Sylvia curruca* Linnaeus, 1758	NA	41	0	0	NA	NA	NA
*Turdus iliacus* Linnaeus, 1766	*Ptilonyssus euroturdi* Fain et Hyland, 1963	27	7	1	NR*	Arkhangelsk Region, Russia	NA
*Turdus iliacus* Linnaeus, 1766	*Ptilonyssus euroturdi* Fain et Hyland, 1963	27	7	2	NR*	Arkhangelsk Region, Russia	NA
*Turdus iliacus* Linnaeus, 1766	*Ptilonyssus euroturdi* Fain et Hyland, 1963	27	7	1	NR*	Arkhangelsk Region, Russia	NA
*Turdus iliacus* Linnaeus, 1766	*Ptilonyssus euroturdi* Fain et Hyland, 1963	27	7	3	NR*	Arkhangelsk Region, Russia	NA
*Turdus iliacus* Linnaeus, 1766	*Ptilonyssus euroturdi* Fain et Hyland, 1963	27	7	2	NR*	Arkhangelsk Region, Russia	NA
*Turdus iliacus* Linnaeus, 1766	*Ptilonyssus euroturdi* Fain et Hyland, 1963	27	7	2	NR*	Arkhangelsk Region, Russia	NA
*Turdus iliacus* Linnaeus, 1766	*Ptilonyssus euroturdi* Fain et Hyland, 1963	27	7	1	NR*	Arkhangelsk Region, Russia	NA
*Turdus merula* Linnaeus, 1758	*Sternostoma dureni* Fain, 1956	78	1	1	NR*	Hervir, Leningrad Region, Russia	NA
*Turdus philomelos* Brehm, 1831	*Sternostoma turdi* Zumpt et Till, 1955	19	7	2	NR, ER WE	Severodvinsk, Arkhangelsk, Russia	Butenko 1965; Fain 1956; Fain 1957; Fain 1959; Fain 1962a; Fain 1963; Fain and Aikten 1967; Fain et al. 1974; Furman 1957; Pence 1972c; Shumilo and Lunkashu 1970; Sixl 1971; Spicer 1984; Spicer 1987; Zumpt and Till 1955
*Turdus philomelos* Brehm, 1831	*Sternostoma turdi* Zumpt et Till, 1955	19	7	2	NR, ER WE	Severodvinsk, Arkhangelsk, Russia	Butenko 1965; Fain 1956; Fain 1957; Fain 1959; Fain 1962a; Fain 1963; Fain and Aikten 1967; Fain et al. 1974; Furman 1957; Pence 1972c; Shumilo and Lunkashu 1970; Sixl 1971; Spicer 1984; Spicer 1987; Zumpt and Till 1955
*Turdus philomelos* Brehm, 1831	*Sternostoma turdi* Zumpt et Till, 1955	19	7	1	NR, ER WE	Severodvinsk, Arkhangelsk, Russia	Butenko 1965; Fain 1956; Fain 1957; Fain 1959; Fain 1962a; Fain 1963; Fain and Aikten 1967; Fain et al. 1974; Furman 1957; Pence 1972c; Shumilo and Lunkashu 1970; Sixl 1971; Spicer 1984; Spicer 1987; Zumpt and Till 1955
*Turdus philomelos* Brehm, 1831	*Sternostoma turdi* Zumpt et Till, 1955	19	7	1	NR, ER WE	Severodvinsk, Arkhangelsk, Russia	Butenko 1965; Fain 1956; Fain 1957; Fain 1959; Fain 1962a; Fain 1963; Fain and Aikten 1967; Fain et al. 1974; Furman 1957; Pence 1972c; Shumilo and Lunkashu 1970; Sixl 1971; Spicer 1984; Spicer 1987; Zumpt and Till 1955
*Turdus philomelos* Brehm, 1831	*Sternostoma turdi* Zumpt et Till, 1955	19	7	1	NR, ER WE	Severodvinsk, Arkhangelsk, Russia	Butenko 1965; Fain 1956; Fain 1957; Fain 1959; Fain 1962a; Fain 1963; Fain and Aikten 1967; Fain et al. 1974; Furman 1957; Pence 1972c; Shumilo and Lunkashu 1970; Sixl 1971; Spicer 1984; Spicer 1987; Zumpt and Till 1955
*Turdus philomelos* Brehm, 1831	*Sternostoma turdi* Zumpt et Till, 1955	19	7	1	NR, ER WE	Severodvinsk, Arkhangelsk, Russia	Butenko 1965; Fain 1956; Fain 1957; Fain 1959; Fain 1962a; Fain 1963; Fain and Aikten 1967; Fain et al. 1974; Furman 1957; Pence 1972c; Shumilo and Lunkashu 1970; Sixl 1971; Spicer 1984; Spicer 1987; Zumpt and Till 1955
*Turdus philomelos* Brehm, 1831	*Sternostoma turdi* Zumpt et Till, 1955	19	7	1	NR, ER WE	Severodvinsk, Arkhangelsk, Russia	Butenko 1965; Fain 1956; Fain 1957; Fain 1959; Fain 1962a; Fain 1963; Fain and Aikten 1967; Fain et al. 1974; Furman 1957; Pence 1972c; Shumilo and Lunkashu 1970; Sixl 1971; Spicer 1984; Spicer 1987; Zumpt and Till 1955
*Turdus pilaris* Linnaeus, 1758	NA	121	0	0	NA	NA	NA
*Dendrocopos major* Linnaeus, 1758	NA	16	0	0	NA	NA	NA
