# Supplementary figures and images for: A comprehensive survey of Rhinonyssid mites (Mesostigmata: Rhinonyssidae) in Northwest Russia: New mite-host associations and prevalence data
Source: Biodivers Data J. 2020 Feb 28;8:e49535. doi: 10.3897/BDJ.8.e49535 (PMC7060284; doi:10.3897/BDJ.8.e49535)

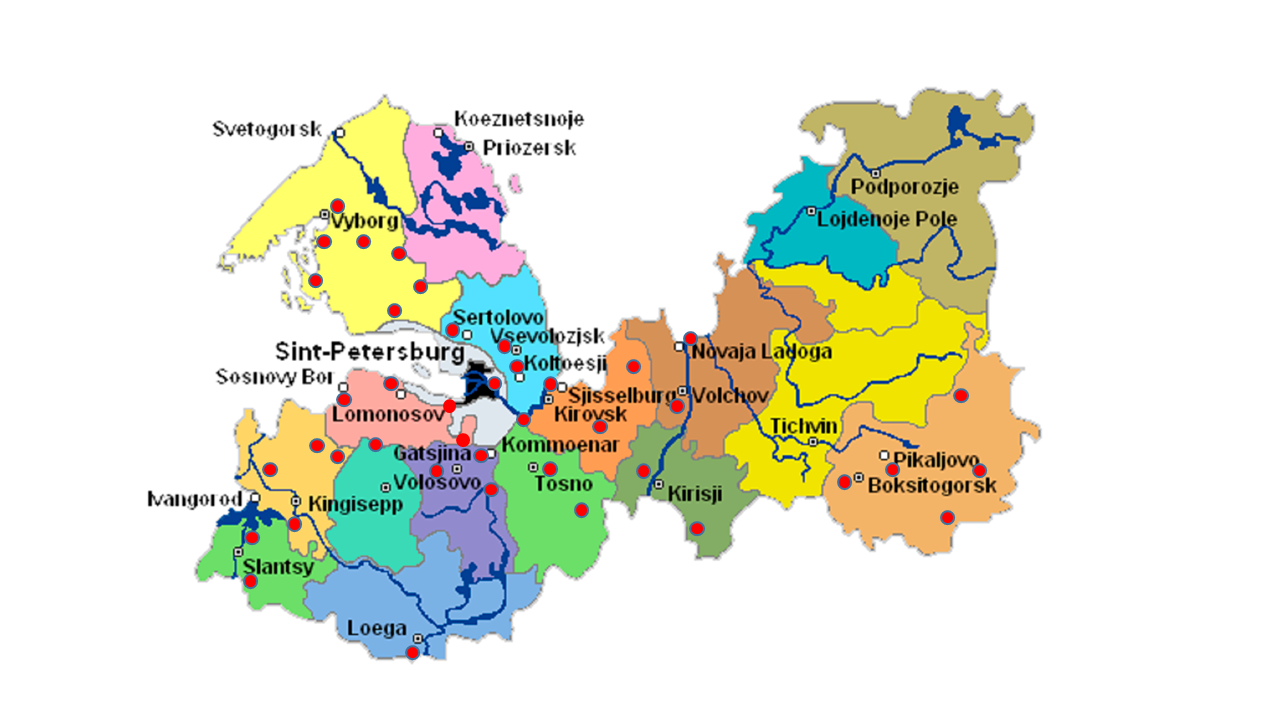

Supplement: Supplementary material 1 — Figure S1 [file bdj-08-e49535-s001.png]
